# Complete mitochondrial genome of *Saiga tatarica* (Ruminantia; Pecora; Bovidae) isolate Wuwei in China

**DOI:** 10.1080/23802359.2017.1383199

**Published:** 2017-09-26

**Authors:** Xin Ding, Jin Wu, Hui Xiao, Zhaojun Wang, Qi Liu, Xuedong Liu, Kun Jin, Dong Zheng

**Affiliations:** a College of Wildlife Resources, Northeast Forestry University, Harbin, China;; b Gansu Endangered Animal Research Center, Wuwei, China;; c Research Institute of Forest Ecology, Environment and Protection, Chinese Academy of Forestry, Beijing, China

**Keywords:** *Saiga tatarica* isolate Wuwei, mitogenome, encoded protein, Trna, rRNA

## Abstract

This report described the complete mitochondrial genome of the Saiga antelope, *Saiga tatarica*, from the Gansu Endangered Animal Research Center (GEARC) in Gansu Province, China. The mitogenome was a circular molecule of 16,376 bps (Genbank accession number: MF497028). It contained 13 protein-coding, 22 tRNA and two rDNA genes. The protein-coding genes had ATA or ATG as the initiation codon, and were terminated by the typical stop codon TAA, except for NAD2 and NAD3. The complete mitogenome sequence would be useful for further understanding origination, evolution and conservation genetics of *S. tatarica* population in China.

The *Saiga* antelope (*Saiga tatarica*) historically distributed in northwest China, and was extirpated in the mid-twentieth century in wild. To prepare for reintroduction plan in the future, a founder herd has been established in the Wuwei Endangered Wildlife Breeding Centre (now called the Gansu Endangered Animal Research Center, GEARC) since 1980s. Currently, the population increased to over 150 individuals (Cui et al. 2017). Due to high mutation rate, mitochondrial DNA information could provide valuable information for (sub)species identification, phylogenetic and captive population genetic analysis, including specifically tracing female lines of descent or migration patterns (Cantatore and Saccone 1987). To our knowledge, the complete mitogenome sequence of *S. tatarica* in China has not been reported.

Sample treatment protocols were approved by the Animal Care Committees at Northeast Forestry University. We extracted genomic DNA from placenta tissue collected and stored at the GEARC (102°53’13”E, 37°53’12”N), and sequenced with HisSeq PE150 (Illumina). The resultant reads were mapped to the reference mitogenome of *Bos taurus* with BWA software (Li and Durbin 2009), and analyzed by SAMTOOLS (Li et al. 2009) and DOGMA web server (Wyman et al. 2004). We used MEGA software to perform Maximum Likelihood analysis based on the complete mitogenome sequence (Tamura et al. 2013). The phylogenetic history was inferred by using the GTR + G + I model, with the highest log likelihood (−77791.0724).

The *Saiga* antelope mitogenome was a 16,376 bps closed loop (accession number MF497028). This represented a typical mammalian mitochondrial genome (Saccone et al. 1999; Gibson et al. 2005), in that it comprised 13 protein-coding, 22 putative tRNA, and two rDNA genes. The average GC content of the *Saiga* antelope mitogenome was 37.78%. Similar to the other bovid mitochondrial genomes, the heavy strand (H-strand) was predicted to contain 12 protein-coding, 14 tRNA genes and two rDNA genes, and the light strand (L-strand) was predicted to contain 1 protein-coding, eight tRNA genes. The genes, ATP8 and ATP6, shared 40 nucleotides, ND4 and ND4L shared 7 nucleotides, and ND5 and NAD6 shared 17 nucleotides. Ten protein-coding genes of the *Saiga* antelope mitogenome started with ATG, NAD2, NAD3 and NAD5 started with ATA. The stop codon in each of these protein-coding genes was either TAA, except for NAD2 and NAD3, which had TAG.

To investigate the phylogenetic relationships within Antilopinae, with *Bos taurus* as outgroup, we analysed complete mitogenome sequences from 26 closely related taxa (Figure 1). The result partially supported the basic topology recoverable from combined mitochondrial and nuclear sequence analyses: gazelles were resolved as paraphyletic; within Antilopina subgroup, *Saiga* was the sister-species to all other species (*Antidorcas*, *Litocranius*, *Antilope* and gazelles); meanwhile the position of the root in Antilopini was still unsettled (Bärmann et al. 2013). The data generated in the present study would help in understanding genetic origination and evolution, further would aid in the conservation of the *Saiga* antelope population in China.

**Figure 1.**
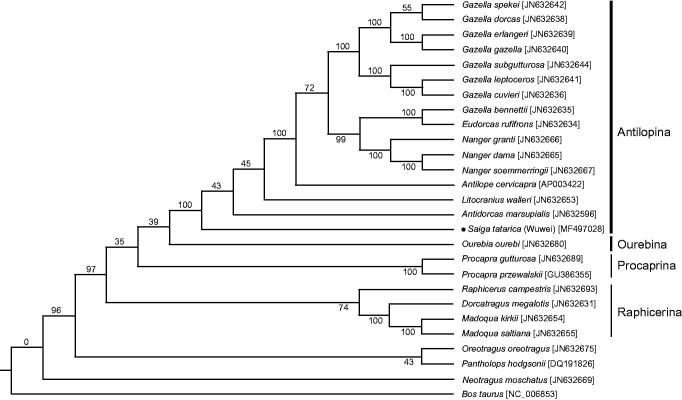
Molecular phylogenetic among 26 members of Antilopinea (Ruminantia; Pecora; Bovidae) based on the complete mitogenome sequences. The evolutionary history was inferred by using the Maximum Likelihood method based on the GTR + G + I model. The numbers beside the nodes are percentages of 1000 bootstrap values. The *Bos taurus* species was used as outgroup. The dot indicated the species in this study. Alphanumeric terms indicate the GenBank accession numbers.

## Geolocation information

Geospatial coordinates for the Saiga antelope placenta collection: 102°53’13”E, 37°53’12”N.
